# Assessment of Anterior Segment Measurements with Swept Source Optical Coherence Tomography before and after Ab Interno Trabeculotomy (Trabectome) Surgery

**DOI:** 10.1155/2016/4861837

**Published:** 2016-10-03

**Authors:** Handan Akil, Ping Huang, Vikas Chopra, Brian Francis

**Affiliations:** ^1^Doheny Eye Institute, Doheny Image Reading Center, Los Angeles, CA, USA; ^2^Department of Ophthalmology, David Geffen School of Medicine, Los Angeles, CA, USA

## Abstract

*Purpose*. To compare the changes of anterior segment parameters, assessed by swept source anterior segment optical coherence tomography (AS-OCT) after combined Trabectome-cataract surgery and Trabectome-only surgery in open angle glaucoma patients.* Methods*. Thirty-eight eyes of 24 patients with open angle glaucoma were scanned with swept source AS-OCT before and 4 weeks after combined Trabectome-cataract or Trabectome-only surgery. Intraocular pressure, number of medications, and AS-OCT parameters, such as angle opening distance at 500 and 750 *μ*m from the scleral spur (AOD500 and AOD750), trabecular-iris space area at 500 and 750 mm^2^ (TISA500, TISA750), angle recess area at 500 and 750 mm^2^ (ARA500, ARA750), trabecular iris angle (TIA), anterior chamber depth (ACD), anterior chamber width (ACW), and anterior chamber volume (ACV), were obtained before the surgery. These parameters were compared to evaluate whether the outcome of the surgery differed among the patients after the surgery. The width of the trabecular cleft was also measured for both groups.* Results*. The reduction of IOP and number of medications was found to be statistically significant in both groups (*p* < 0.001). ACD, ACV, and angle parameters such as AOD 500/750, TISA 500/750, ARA 500/750, and TIA500 showed significantly greater changes from the preoperative values to postoperative 1st month values in combined Trabectome-cataract surgery group (*p* < 0.05), whereas Trabectome-only group did not show statistically significant difference (*p* > 0.05). There was no statistically significant difference between two groups for the width of the trabecular cleft (*p* = 0.7).* Conclusion*. Anterior chamber angle parameters measured with swept source AS-OCT may be useful for evaluating glaucoma patients before and after Trabectome surgery with or without cataract surgery.

## 1. Introduction

Glaucoma is one of the leading causes for irreversible blindness worldwide [1]. To date, lowering the intraocular pressure (IOP) has been known to be the only proven therapy to slow the progression of optic nerve damage. Classic glaucoma filtration surgery by means of trabeculectomy or glaucoma aqueous shunt implants is the most effective way of lowering intraocular pressure in the long term. Although both surgical procedures are well established, potentially dangerous intraoperative and postoperative complications are known to occur [2]. This has led to the development of the concept of minimally invasive glaucoma surgery (MIGS). This surgical approach does not alter the conjunctiva or involve an external filtration bleb, as it enhances the traditional trabecular outflow pathway [3]. It has been previously reported that trabeculotomy by internal approach (Trabectome: NeoMedix Corporation, Tustin, CA, USA) surgery performed with or without cataract surgery can lower intraocular pressure and dependence on glaucoma medications while maintaining an excellent safety profile [3, 4]. The surgical procedure involves the selective removal of the inner wall of Schlemm's canal and the juxtacanalicular trabecular meshwork, which is theorized to be the source of the most resistance to aqueous humor outflow [5]. Ablation of these areas using the Trabectome without damaging the collector channels facilitates aqueous outflow and reduces IOP. This surgical method can be performed through a clear corneal incision, and the primary sites of abnormal flow resistance can be removed without damaging the conjunctiva [4].

Optical coherence tomography (OCT) has become an essential tool in ophthalmic imaging, which provides objective documentation of the angle [6, 7], as opposed to the subjective evaluation using gonioscopy. OCT technology has been improved from time-domain to spectral-domain systems, with enhanced image acquisition speed and resolution [8]. Spectral-domain systems yield A-scan rates from 20 kHz [9] to 400 kHz (swept source) [10] and are capable of 3-dimensional imaging of biological structures. There are only a few commercially available spectral-domain OCT (SD-OCT) devices that are able to image the anterior segment. Swept source AS-OCT (SS-OCT, CASIA SS-1000, Tomey Corporation, Nagoya, Japan) is a variation of spectral-domain OCT that is specifically designed for anterior segment imaging using 1,310 nm wave width with a scan speed of 30,000 A-scans per second and an axial resolution of less than 10 *μ*m. In the high-definition scan mode, both scleral spur (SS) and Schwalbe's line (SL) can be identified [11–13].

Therefore, because of the ability of SS-OCT to accurately and reproducibly characterize anterior chamber structures, the aim of this prospective study was to evaluate the changes of the anterior chamber parameters before and after combined Trabectome-cataract and Trabectome-only surgery using swept source AS-OCT.

## 2. Methods

This prospective comparison study was approved by the local ethics committee and followed the regulations of the Declaration of Helsinki. After written informed consent, a total of 38 eyes of 24 patients (18 eyes in Trabectome-only surgery group, 20 eyes in combined Trabectome-cataract surgery group) with primary open angle glaucoma (POAG) were included in the study in a consecutive manner from August 2015 to February 2016 at the Doheny Eye Institute, UCLA.

All subjects underwent comprehensive ophthalmic examinations, including measurement of the best-corrected visual acuity, Goldmann applanation tonometry, slit-lamp biomicroscopy, gonioscopy, dilated fundus examination, OCT, and standard automated perimetry, using the Humphrey Visual Field Analyzer (Carl Zeiss, Inc., Dublin, CA, USA) and integrated 24-2 Swedish Interactive Threshold Algorithm (SITA) software program.

Primary open angle glaucoma was defined as the presence of glaucomatous optic nerve damage (localized or diffuse neuroretinal rim thinning and/or retinal nerve fiber layer defect) and an associated visual field defect, with an open angle confirmed on gonioscopy in the absence of other known causes of glaucomatous disease. Mean Cup/Disk ratio was 0.7 ± 0.2, visual field mean deviation (MD) was −5.6 ± 4.9 dB, and visual field pattern standard deviation (PSD) was 4.3 ± 4.7 dB.

Swept source AS-OCT (SS-1000 CASIA; Tomey Co. Ltd. Nagoya, Japan) was performed before and 1 month after combined Trabectome-cataract and Trabectome-only surgery. The same surgeon (BF) performed trabeculotomy using Trabectome first and then phacoemulsification, if indicated, according to the surgical technique as described in previous studies [3, 5].

All eyes were imaged without dilation (nonmydriasis) in the room light (light intensity, 368 lux) by a trained glaucoma research fellow (HA) using the “3D-angle high definition (HD)” protocol, which was composed of a volumetric scan (dimension, 8 mm × 4 mm and 64 B scans) and this allowed 360-degree imaging of the whole anterior segment in 2.4 seconds. The seated subject fixated at an internal fixation target during the imaging. To avoid lid artifact, participants were instructed to pull down the lower lid against the lower orbital rim to expose the lower limbus while the imager elevated the upper lid against the upper orbital rim to expose the upper limbus. All images obtained were free from lid artifacts. A total of 128 cross-sectional images of the anterior chamber were collected. The semiautomated software of the CASIA can be used to measure the anterior chamber angle parameters. For the quantitative assessment of the anterior segment parameters, prior identification of a landmark, scleral spur (SS), is necessary. The SS is identified as the point where there is a change in curvature of the corneoscleral interface in two-dimension analysis of software on the chosen horizontal frame. After the scleral spur and trabecular cleft were identified manually on nasal sample slices, the customized analytic software exported the values of anterior chamber parameters of that slice.

The anterior chamber parameters given automatically by swept source AS-OCT, angle opening distance at 500–750 mm (AOD 500/750), trabecular-iris space area at 500–750 mm (TISA 500/750), angle recess area at 500–750 mm (ARA500/750), trabecular iris angle (TIA 500/750) at surgical quadrants, anterior chamber volume (ACV), anterior chamber width (ACW: from spur to spur), and trabecular cleft width, were incorporated for analysis [14–17].

**Figure 1:**
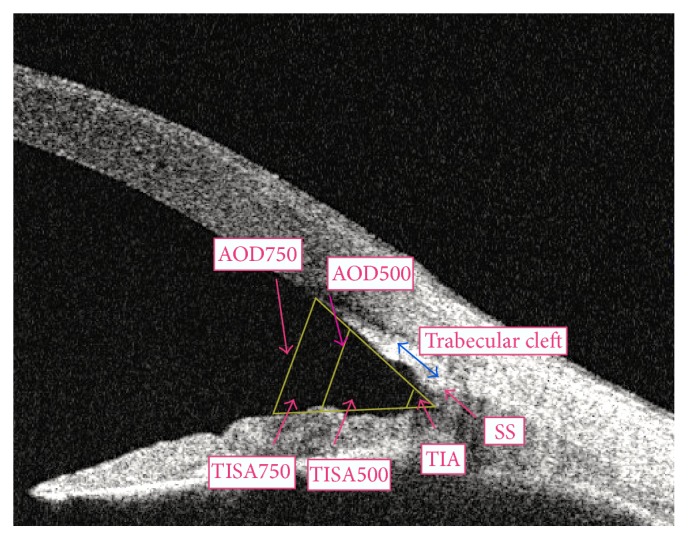
A cross-sectional OCT image of the nasal angle showing the anterior chamber angle parameters of a patient in combined Trabectome group postoperatively.

Images were analyzed with the Angle Assessment Program (CASIA software). After the scleral spurs are identified, the software calculates various parameters of the iris, cornea, and lens using automated identification of the anterior and posterior surfaces of the cornea, iris, and the anterior surface of the lens. We were also able to measure the width of the trabecular cleft with the same software in manual mode. Good quality images of the angle area provided assessment of the surgical site and other anterior chamber parameters (Figure 1). Ten eyes underwent scanning two times for image acquisition reproducibility analysis. All images were analyzed by 2 research fellows (HA and PH) for intra- and intergrader repeatability analysis. Intraclass correlation coefficient (ICC) was calculated for all the repeatability.

## 3. Statistical Analysis

Statistical analysis was performed using SPSS software version 18 (SPSS, Inc., Chicago, IL, USA). The mean and SD were calculated for the anterior segment variables. Continuous variables were analyzed using Wilcoxon signed rank test. The anterior segment parameters were compared before and after the surgery. For comparison between the 2 groups, repeated measure ANOVA was used. A *p* value less than 0.005 was considered statistically significant.

## 4. Results

The mean age was 76.32 ± 9.9 years in the combined Trabectome-cataract surgery group (men: 11 eyes, women: 9 eyes) and 72.5 ± 7 years in the Trabectome-only group (men: 8 eyes, women: 10 eyes). There were no significant intraoperative or postoperative complications. Mean age did not differ significantly between the groups (*p* > 0.05).

The interobserver agreement for assessment of visibility of the angle structures of two groups was high with agreement coefficients ranging between 0.82 and 0.987.

For the Trabectome-only group, the mean preoperative IOP of 24.2 ± 4.7 mm Hg was reduced by 40% to 14.6 ± 3.2 mm Hg at the 1st month. For combined Trabectome-cataract group, the mean preoperative IOP of 25.3 ± 6.4 mm Hg was reduced by 44% to 14.2 ± 2.8 mm Hg at the 1st month.

Medications decreased from 2.6 ± 1.2 to 1.7 ± 1.2 with a 32% reduction in combined Trabectome-cataract group and from 2.9 ± 1.2 to 2.1 ± 1.5 with a 28% reduction in Trabectome-only group.

There is statistically significant decrease of IOP and number of medications in both groups after the surgery (*p* < 0.001). But no statistically significant difference was found between the two groups in amount of IOP reduction and number of medications (*p* > 0.05).

There was no significant difference for the preoperative baseline anterior segment anatomy values of both groups (*p* > 0.05). Trabectome-only group did not show a statistically significant difference between preoperative and postoperative measurements of anterior chamber angle (ACA) parameters (*p* > 0.05). ACD, ACV, and all the angle parameters such as AOD 500/750, TISA 500/750, ARA 500/750, and TIA500 of the combined Trabectome-cataract surgery group were significantly different from the preoperative values (*p* < 0.05). ACD and TIA500 values showed significantly greater changes from the preoperative values to postoperative 1st month values in combined Trabectome-cataract surgery group compared to the Trabectome-only group (*p* < 0.001), whereas the ACW value was not significantly different. The mean differences of ACW, ACD, and ACV were 0.24 ± 0.2, 0.5 ± 0.11, and 26.65 ± 8.8, respectively (*p* = 0.25, *p* < 0.001, and *p* = 0.05) in the combined Trabectome-cataract surgery group (Table 1) and 0.26 ± 0.12, 0.12 ± 0.05, and 2.26 ± 1.8 (*p* = 0.2, *p* = 0.11, and *p* = 0.8) in Trabectome-only group (Table 2). The width of the trabecular cleft was 0. 358 ± 0.13 mm in the combined Trabectome-cataract group and was 0. 342 ± 0.1 mm in the Trabectome-only group. There was no statistically significant difference between two groups for the width of the trabecular cleft (*p* = 0.7). ICC values for intragrader reproducibility, intergrader reproducibility, and repeatability measures were found excellent (Table 3).

## 5. Discussion

Ab interno trabeculectomy using the Trabectome is a relatively new option for reducing the IOP and the number of glaucoma medications in selected patients with primary and secondary open angle glaucoma [5]. The present study showed that there is a statistically significant decrease in the IOP and number of medications after both stand-alone and combined surgeries comparable to previous studies [3, 5, 6].

Surgical intervention is an important aspect of glaucoma management. Pre- and postoperative angle imaging are useful for documenting and evaluating surgery outcomes. Gonioscopy as a traditional method is used in the visualization of anterior chamber angle; however, it is a contact method and has certain limitations such as high interobserver variability and difficulty in quantification of the angle [7–10]. AS-OCT can be used for noncontact measurement even immediately following surgery without the risk of infection or wound dehiscence. Thus, early postoperative analysis is possible by measuring data starting on the day after surgery. In the present study, there were no cases in which identifying the SS was difficult (Figure 1). In addition, we were able to evaluate the trabecular cleft and measure the width easily after both of the surgeries.

**Figure 2:**
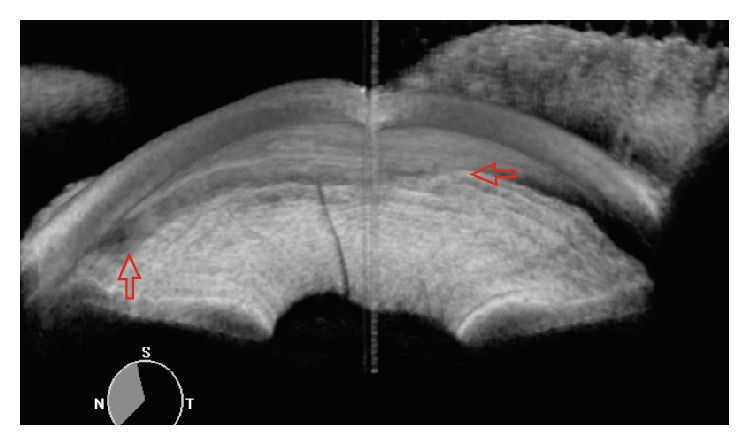
A nasal quadrant screenshot of the video gonioscopy of the post-Trabectome patient which gives information about the trabecular cleft of the angle morphology in high resolution circumferentially in 360 degrees. Red arrows show the Trabectome surgical site.

An advantage of using swept source AS-OCT for postoperative assessment is its short scan time. Less than 3 seconds is required to image the angle morphology in high resolution circumferentially in 360 degrees (Figure 2). Another advantage of swept source AS-OCT imaging is its ability to discern the patency of trabecular cleft, which sometimes is difficult to be visualized with gonioscopy.

The swept source AS-OCT device is useful for detailed angle evaluation and the imaging procedure is convenient, imposing fewer difficulties on the patients, and can be performed in a shorter time [18]. However, although the interobserver variability is small [19], identifying the scleral spur (SS) can be difficult with other spectral-domain OCT devices.

**Figure 3:**
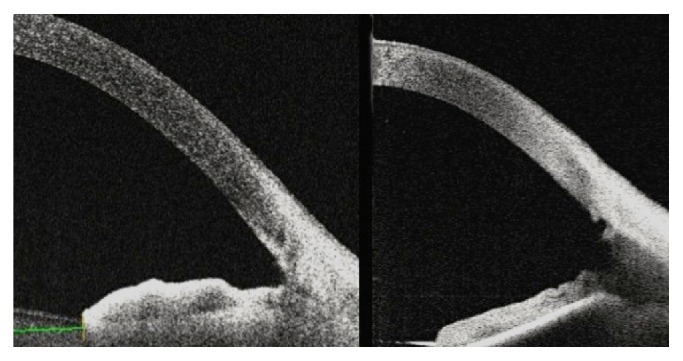
A cross-sectional OCT image of the nasal angle following combined Trabectome-cataract surgery. This frame-averaged image shows that the posterior trabecular meshwork has been removed, leaving a wide trabecular cleft.

**Figure 4:**
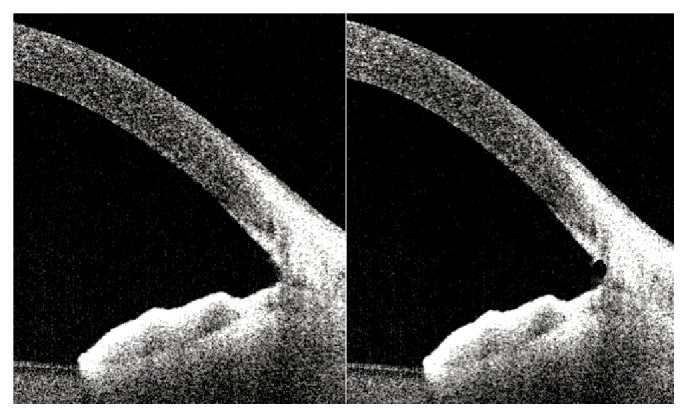
A cross-sectional OCT image of the nasal angle following Trabectome-only surgery. This frame-averaged image shows that the posterior trabecular meshwork has been removed.

Many studies have shown that distance-based measurements made 500 *μ*m and/or 750 *μ*m from the scleral spur are clinically valid for the evaluation of the ACAs [20–22]. These distance-based parameters, such as AOD, are usually based on the assumption that the iris surface conforms to a straight line, but irregularity of iris contour and curvature are commonplace. To overcome this limitation, researchers have devised alternative area-related parameters, such as angle recess area (ARA) and TISA. Both parameters have shown good discrimination between open and narrow ACAs when compared to grading using gonioscopy [20–22]. Our patients who had combined Trabectome-cataract surgery showed statistically significant increases in AOD, TISA, TIA, ACD, and ACV measurements. The angle parameters of the Trabectome-only group did not increase to the same extent as the angle parameters in combined Trabectome-cataract group. AOD 500 measurement might be affected by the cleft depth and therefore ARA and TISA metrics may be clinically more reliable for postoperative evaluation. Additionally, the current study suggests that combined Trabectome-cataract surgery affects the angle parameters more with lens extraction by means of increasing the space between the iris and cornea (Figures 3 and 4).

Liu et al. showed that swept source AS-OCT provides reproducible measurement of AOD, TISA, and TIA at different meridians, suggesting that it is useful for measuring the angle for risk assessment and for evaluating longitudinal changes before and after therapeutic intervention in patients. They found an association between the angle width and the variances of AOD and TISA [23].

Lee et al. also studied the angle parameters before and after cataract surgery in an Asian population and found that swept source OCT is useful for the evaluation of the changes in angle parameters before and after the cataract surgery [24].

There are also some studies which have focused on the changes of the anterior chamber angle in glaucomatous and healthy eyes using various technologies. Zou et al. showed no statistically significant difference of ACV, ACD, and ACA by Pentacam Scheimpflug system between open angle patients and age-matched healthy controls [25]. Cataract extraction and IOL implantation can significantly deepen the ACD, widen the anterior chamber drainage angle, and lower IOP in narrow angle and open angle eyes [26]. Although ACA, ACD, and ACV were found to be increased a little less in open angle glaucoma than in narrow angle glaucoma, the difference was still found statistically significant after uncomplicated phacoemulsification [27, 28].

To the best of our knowledge, this is the first study that shows how to evaluate anterior chamber parameters with a trabecular cleft by swept source AS-OCT after the combined Trabectome-cataract and Trabectome-only surgery. Clinical significance of comparing the changes of anterior angle parameters after Trabectome surgery in narrow angle glaucoma would be expected easily, so we only recruited patients with open angle glaucoma.

Importantly, our data suggest that evaluation and followup of Trabectome surgical sites with swept source OCT imaging may help us understand the reason for the success and failure of the surgery with the evidence for excellent repeatability and intra- and intergrader reproducibility. The relation between the structural change of the trabecular cleft and success rate of the Trabectome surgery may be a hypothesis for future research.

Several limitations of our study warrant mention. First of all, in the current study, the number of patients is low. Secondly, it would be better to have a control group which undergoes only cataract surgery. One may argue that angle measurements using Schwalbe line would help clinicians more but the device does not have software based on that. Another limitation could be related to lightning condition of the study. The imaging procedure was performed in room light (without dilation) to evaluate the changes of the angle structures precisely after the surgery but swept source OCT is usually done in complete dark room to maintain standard light conditions. This can lead to variations and may not be comparable to other similar studies in which imaging procedures were done in complete dark conditions. Additionally, the current study evaluated short-term effects of Trabectome on the angle structures of open angle patients but follow-up assessment with swept source OCT in longer terms might be more helpful for both open and closed angle patients. It should be noted that the success of the surgical procedures is not the subject of this report.

Over the past years, the technology of OCT has evolved very rapidly. Commercially available SD-OCT devices use a shorter wavelength and allow higher scanning speed and increased axial and transverse resolution. Despite the fact that shadowing artifacts and decreased ability to image the scleral spur can still occur with this technology, the improvement in image resolution has provided a more detailed analysis of angle structures. The present study showed that scleral spur visibility was excellent and placement of measurement tools was reproducible with swept source OCT technology. But this might also result from the fact that images were acquired by an ophthalmologist who checked image quality after the acquisition.

In conclusion, imaging the anterior chamber angle with swept source OCT may provide an efficient approach to visualize the anterior chamber before and after Trabectome surgery. Measurement of the angle parameters and cleft width longitudinally with success rates may be useful to detect angle and cleft pathology in relation to IOP increase and glaucoma progression.

## Figures and Tables

**Table 1:** Comparison of the angle measurements with swept source OCT before and after Trabectome-only surgery.

Variables	Preoperative	Postoperative	Mean difference	*p* value^*∗*^
AOD 500 [mm]	0.51 ± 0.34	0.62 ± 0.289	0.11 ± 0.1	0.3
AOD 750 [mm]	0.68 ± 0.37	0.7 ± 0.23	0.02 ± 0.01	0.8
TISA 500 [mm^2^]	0.186 ± 0.04	0.21 ± 0.03	0.02 ± 0.05	0.09
TISA 750 [mm^2^]	0.334 ± 0.2	0.332 ± 0.15	−0.002 ± 0.11	0.9
TIA 500 [°]	45.6 ± 2.27	46.12 ± 3.16	1.06 ± 0.32	0.6
ARA 500 [mm^2^]	0.26 ± 0.02	0.28 ± 0.04	0.02 ± 0.003	0.06
ARA 750 [mm^2^]	0.44 ± 0.15	0.48 ± 0.2	0.04 ± 0.05	0.5
ACW [mm]	11.95 ± 0.63	12.21 ± 0.51	0.26 ± 0.12	0.2
ACD [mm]	2.96 ± 0.26	3.08 ± 0.16	0.12 ± 0.05	0.11
ACV [mm^3^]	168.64 ± 45.63	170.9 ± 15.68	2.26 ± 1.8	0.8

Mean ± standard deviation.

^*∗*^Wilcoxon signed rank test, *p* < 0.005 statistically significant.

**Table 2:** Comparison of the angle measurements with swept source OCT before and after combined Trabectome-cataract surgery.

Variables	Preoperative	Postoperative	Mean difference	*p* value^*∗*^
AOD 500 [mm]	0.504 ± 0.254	0.71 ± 0.26	0.21 ± 0.12	0.015
AOD 750 [mm]	0.72 ± 0.263	0.88 ± 0.24	0.16 ± 0.02	0.05
TISA 500 [mm^2^]	0.196 ± 0.112	0.296 ± 0.143	0.09 ± 0.03	0.03
TISA 750 [mm^2^]	0.349 ± 0.17	0.478 ± 0.22	0.13 ± 0.06	0.04
TIA 500 [°]	43.4 ± 4.65	51.2 ± 5.32	7.8 ± 1.58	<0.001
ARA 500 [mm^2^]	0.2 ± 0.1	0.29 ± 0.15	0.09 ± 0.04	0.03
ARA 750 [mm^2^]	0.43 ± 0.25	0.58 ± 0.24	0.15 ± 0.07	0.04
ACW [mm]	11.73 ± 0.58	11.97 ± 0.72	0.24 ± 0.2	0.25
ACD [mm]	3.1 ± 0.38	3.62 ± 0.31	0.5 ± 0.11	<0.001
ACV [mm^3^]	159.634 ± 36.77	186.29 ± 14.3	26.65 ± 8.8	0.005

Mean ± standard deviation.

^*∗*^Wilcoxon signed rank test, *p* < 0.005 statistically significant.

**Table 3:** Intraobserver and interobserver reproducibility and repeatability measurement of angle parameters with the swept source OCT.

	Intergrader ICC (95% CI)	Intragrader ICC (95% CI)	Repeatability ICC (95% CI)
SS-AOD 500	0.927 (0.896–0.949)	0.958 (0.930–0.975)	0.977 (0.966–0.984)
SS-AOD 750	0.936 (0.909–0.956)	0.931 (0.885–0.959)	0.970 (0.957–0.979)
SS-TISA 500	0.915 (0.875–0.933)	0.912 (0.856–0.947)	0.964 (0.948–0.975)
SS-TISA 750	0.869 (0.833–0.945)	0.843 (0.749–0.904)	0.957 (0.938–0.970)
Trabecular cleft width	0.960 (0.938–0.975)	0.940 (0.920–0.950)	0.946 (0.940–0.960)

SS: scleral spur, AOD: angle opening distance, TISA: trabecular-iris space area, ICC: intraclass correlation coefficient, and CI: confidence interval.
